# Heart Valve Involvement in Patients with Antiphospholipid Syndrome: A Long-Term Follow-Up Study of a Single Centre

**DOI:** 10.3390/jcm12082996

**Published:** 2023-04-20

**Authors:** Isaac Pons, Joana Louro, Marta Sitges, Bàrbara Vidal, Ricard Cervera, Gerard Espinosa

**Affiliations:** 1 Department of Autoimmune Diseases, Reference Centre (UEC/CSUR) for Systemic Autoimmune Diseases of the Catalan and Spanish Health Systems, Hospital Clínic, Universitat de Barcelona, 08036 Barcelona, Spain; ipons@althaia.cat (I.P.); louro_joana@yahoo.com (J.L.); gespino@clinic.cat (G.E.); 2 Institut d’Investigacions Biomèdiques August Pi i Sunyer (IDIBAPS), 08036 Barcelona, Spain; msitges@clinic.cat (M.S.); bvidal@clinic.cat (B.V.); 3 Department of Internal Medicine, Althaia Xarxa Assistencial Universitària de Manresa, Facultat de Medicina, Universitat de Vic-Universitat Central de Catalunya (UVic-UCC), 08240 Manresa, Spain; 4 Institut Clínic Cardiovascular (ICCV), Hospital Clínic, Universitat de Barcelona, 08036 Barcelona, Spain; 5 Centro de Investigación Biomédica en Red Enfermedades Cardiovasculares (CIBERCV), 28029 Madrid, Spain

**Keywords:** valve involvement, valvulopathy, antiphospholipid syndrome, antiphospholipid antibodies, lupus anticoagulant

## Abstract

Background: Valve involvement is the most common cardiac manifestation in antiphospholipid syndrome (APS). The objective of the study was to describe the prevalence, clinical and laboratory features, and evolution of APS patients with heart valve involvement. Methods: A retrospective longitudinal and observational study of all APS patients followed by a single centre with at least one transthoracic echocardiographic study. Results: 144 APS patients, 72 (50%) of them with valvular involvement. Forty-eight (67%) had primary APS, and 22 (30%) were associated with systemic lupus erythematosus (SLE). Mitral valve thickening was the most frequent valve involvement present in 52 (72%) patients, followed by mitral regurgitation in 49 (68%), and tricuspid regurgitation in 29 (40%) patients. Female sex (83% vs. 64%; *p* = 0.013), arterial hypertension (47% vs. 29%; *p* = 0.025), arterial thrombosis at APS diagnosis (53% vs. 33%; *p* = 0.028), stroke (38% vs. 21%; *p* = 0.043), livedo reticularis (15% vs. 3%; *p* = 0.017), and lupus anticoagulant (83% vs. 65%; *p* = 0.021) were more prevalent in those with valvular involvement. Venous thrombosis was less frequent (32% vs. 50%; *p* = 0.042). The valve involvement group suffered from higher mortality (12% vs. 1%; *p* = 0.017). Most of these differences were maintained when we compared patients with moderate-to-severe valve involvement (*n* = 36) and those with no or mild involvement (*n* = 108). Conclusions: Heart valve disease is a frequent manifestation in our cohort of APS patients and is associated with demographic, clinical and laboratory features, and increased mortality. More studies are needed, but our results suggest that there may be a subgroup of APS patients with moderate-to-severe valve involvement with its own characteristics that differs from the rest of the patients with mild valve involvement or without valve involvement.

## 1. Introduction

The heart is one of the major target organs of antiphospholipid syndrome (APS), and valvular involvement is the most common cardiac manifestation [1], with a reported prevalence of 11.6% in large cohorts of patients with APS [2]. During the 10-year follow-up study of this cohort, 4.6% of the patients developed previously unknown thickening and/or valve dysfunction [3].

In patients with systemic lupus erythematosus (SLE) and/or APS, heart valve involvement has been related with a higher prevalence of arterial thrombotic events, mainly stroke, concomitant presence of cardiovascular risk factors, livedo reticularis, epilepsy, and migraine [4,5]. Furthermore, recent studies suggest an association between valvular involvement and a specific antiphospholipid antibodies (aPL) profile or titre [6,7,8,9,10,11,12].

In recent years, several prospective studies have analysed the evolution of valve involvement in patients with aPL [4,7,13,14,15,16]. In those performed on patients with primary APS [13,14,15], valve lesions may appear, improve, worsen, or remain unchanged over time regardless of the clinical presentation, analytical profile, or treatment.

There is no consensus on the treatment of valve involvement in APS [17]. Anticoagulation is recommended in patients with thromboembolic episodes attributed to valve disease [18]. However, this recommendation is based only in case series and not for the treatment of the valve dysfunction itself. Several studies have failed to demonstrate the efficacy of any treatment to reverse established lesions or prevent their development [4,7,11,13,14,15,16]. Although there are few case reports describing the successful treatment with corticosteroids and/or immunosuppression, currently their administration is not recommended for valve involvement [19,20].

The aim of our study was to evaluate the prevalence and type of heart valve involvement in patients with APS followed in a tertiary hospital. In addition, we analysed possible associations between valve involvement and demographics, clinical manifestations, and laboratory features.

## 2. Materials and Methods

In this retrospective, longitudinal, and observational study, we have included all APS patients followed in the department of Autoimmune Diseases in Hospital Clínic (Barcelona, Spain), a tertiary referral centre, from January 1980 to December 2014 in which at least one transthoracic echocardiography was performed. All patients fulfilled the current classification criteria for APS [21].

Demographic data at APS diagnosis and cardiovascular risk factors [22] were collected. Data considering APS, thrombotic manifestations, and obstetric morbidity, as well as the different clinical characteristics associated with APS but defined as non-criteria in the international consensus of 2006 [21], were also collected. The type of thrombosis (arterial, venous, and thrombotic microangiopathy) and localization were recorded. All these data were collected from the medical record of each patient and entered in a database designed specifically for this study. Time of follow-up was defined as the time (in months) from diagnosis of APS to the last visit or death.

Results of aPL determination at the APS diagnosis were included. Lupus anticoagulant (LAC) was determined following the specific recommendations of the International Society of Thrombosis and Haemostasis (ITHS) [23,24], and IgG and IgM isotypes of anticardiolipin (aCL) and antiβ2-glycoprotein I antibodies (aβ2GPI) were measured using solid-phase standardised immunoassays (ELISA). Antinuclear antibodies (ANA) were measured by indirect immunofluorescence (IIF) on rodent liver cells and/or HEp-2 cells; anti-double-stranded DNA (dsDNA) antibodies were measured by ELISA and/or IIF on *Crithidia luciliae,* and antibodies against extractable nuclear antigen (ENA: Ro60/SSA, La/SSB, Sm and U1-RNP) by ELISA.

We registered the first echocardiography in all patients and the last in those with valvular involvement at baseline. The echocardiographic protocol and the definitions of the main valvular findings were the same as those previously published by our group [7], where the thickness of the normal mitral and tricuspid valves was between 0.7 and 3 mm, and the normal aortic valve thickness was 0.7–2 mm. Abnormal valvular thickening was therefore considered to be present when a thickness of >3 mm (for the mitral and tricuspid valves) or >2 mm (for the aortic valve) was observed. Valvular vegetation was defined as an abnormal localised echodensity with well-defined borders that was either part of or adjacent to valve leaflets, the subvalvular apparatus, or the great vessels.

The severity stratification of valve disease was done according to the 2003 recommendations for evaluation of the severity of native valvular regurgitation with two-dimensional and Doppler echocardiography, from the American Society of Echocardiography [25], and the 2017 ESC/EACTS Guidelines for the management of valvular heart disease [26].

The treatment prescribed after APS diagnosis was also recorded. During the evolution, data concerning thrombotic relapses, and/or obstetric morbidity, treatment modifications, bleeding complications, persistence of aPL profile, and outcome were collected.

The study was conducted in accordance with the Declaration of Helsinki and approved by the Ethics Committee of Hospital Clinic, Barcelona (protocol code HCB/2018/1221 and date of approval 13 December 2018). Patient consent was waived due to the retrospective nature of this study.

### Statistical Analysis

Categorical data are summarised as percentages; significant differences or associations were analysed using the X^2^ test or Fisher’s exact tests. Continuous variables are presented as mean ± standard deviation (SD) or median (interquartile range, IQR), depending on normality as demonstrated by the Kolmogorov-Smirnov test.

Associations of quantitative data were analysed with Student’s *t*-test and with the non-parametric Mann-Whitney U-test. A two-tailed value of *p* < 0.05 was taken to indicate statistical significance. Statistical analysis was performed using the *SPSS* program (SPSS Statistics 21.0, IBM Corp., Armonk, NY, USA).

## 3. Results

Overall, we identified 144 APS patients for which at least one transthoracic echocardiography was performed, representing 47.3% of the overall cohort of APS patients followed by our centre. One hundred and five patients (72.9%) suffered from primary APS; 36 (25%) had SLE-associated APS; and three (2,1%) patients had Sjögren’s syndrome.

Seventy-two (50%) patients presented with heart valve involvement. The prevalence of valve involvement was similar between primary APS patients and those with SLE (46% vs. 61%; *p* = 0.242). Among APS patients with valve involvement, 48 (67%) patients had primary APS; 22 had SLE (30%), and two had Sjögren’s syndrome-associated APS (3%).

The main demographic characteristics, APS-related clinical manifestations, and laboratory features according to the presence of valvular involvement are shown in Table 1 and Table 2, respectively.

In patients with valve involvement, female sex (83% vs. 64%; *p* = 0.013), arterial hypertension (47% vs. 29%; *p* = 0.025), arterial thrombosis at APS diagnosis (53% vs. 33%; *p* = 0.028), stroke (38% vs. 21%; *p* = 0.043), livedo reticularis (15% vs. 3%; *p* = 0.017), LAC (83% vs. 65%; *p* = 0.021), and double aPL positivity (61% vs. 39%; *p* = 0.045), were more prevalent than in patients without valve involvement. Conversely, thrombosis in venous vessels was less frequent in patients with valve involvement (32% vs. 50%; *p* = 0.042). Having positive ANA 81% vs. 60%; *p* = 0.006), low levels of C4 (29% vs. 14%; *p* = 0.042), and an isolated aCL as aPL profile at APS diagnosis (35% vs. 17%; *p* = 0.013) were the most frequent laboratory findings in the group without valvular involvement. No different frequency of valve involvement was found between primary APS or SLE-associated APS. No differences were found in the treatments received, number of thrombotic recurrences and/or the bleeding events during follow-up between the two groups (Table 1). Of note, valve involvement was associated with higher mortality (12% vs. 1%; *p* = 0.017). The causes of death of nine patients with heart valve involvement was a thrombosis in three of them (myocardial infarction, prosthetic mitral valve thrombosis, and cerebrovascular accident with haemorrhage). In the remaining cases, the causes of death were cardiogenic shock, alveolar haemorrhage, lymphoma, pancreatitis, septic shock of cutaneous origin, and post-surgical hemoperitoneum. The only patient without valve involvement who died presented with catastrophic APS.

In the group of APS patients with heart valve involvement, no statistically significant differences were found when comparing the demographic characteristics, cardiovascular risk factors and APS related clinical manifestations between patients with primary APS and those with SLE-associated APS.

Considering the valvular findings at the first echocardiography, mitral thickening was the most frequent abnormality that was present in 52 (72%) patients, followed by mitral regurgitation in 49 (68%), and tricuspid regurgitation in 29 (40%), respectively. In most of them, valvular regurgitation was mild (Table 3). Moreover, we found no differences in the type of valve involvement between primary APS patients and those with SLE-associated APS (Supplementary Table S1).

Of note, non-bacterial thrombotic endocarditis appeared in 26 (36%) patients, 21 of them on the mitral valve and the remaining 5 on the aortic valve. During follow-up, 12 (17%) of these patients required valvular replacement surgery, ten (83%) of the mitral valve, one (8.5%) of the aortic valve and one (8.5%) of both.

Of the patients with non-bacterial thrombotic endocarditis, four died during follow-up, one due to diffuse alveolar hemorrhage six years after the non-bacterial thrombotic endocarditis found in the initial echocardiogram had disappeared in successive echocardiograms. Another was due to valve thrombosis two months after mechanical mitral valve replacement. A third died due to cardiogenic shock 12 years after mitral valve replacement. The last one, who required a second mitral valve replacement due to prosthetic thrombosis one year after the first mechanical valve prosthesis, died five years after the last intervention due to a hemorrhagic stroke.

In addition, we compared the subgroup of patients with significant valve involvement, including those patients with moderate and severe valvular regurgitation and those with non-bacterial thrombotic endocarditis (*n* = 36 patients), versus those patients without valve involvement and mild valve involvement or valve thickening without functional impact (*n* = 108 patients) (Table 4).

Patients with significant valve involvement had a higher prevalence of arterial hypertension (56% vs. 31%; *p* = 0.016), and they had more arterial thrombosis (75% vs. 32%; *p* < 0.001), stroke (61% vs. 18%; *p* < 0.001), and livedo reticularis (22% vs. 5%; *p* = 0.019), but less venous thrombosis (11% vs. 51%; *p* < 0.001) and pulmonary embolism (3% vs. 18%; *p* = 0.026). Considering aPL profile, LAC (92% vs. 68%; *p* = 0.007) and double aPL positivity (75% vs. 44%; *p* = 0.017) were more frequent in patients with significant valve involvement, persisting in the pattern of double aPL positivity as the most frequent form of presentation. We did not find differences in the treatment they received, the number of thrombotic recurrences, the risk of bleeding, and/or mortality during follow-up between the two groups.

During follow-up, echocardiography was repeated in 40 out of 72 (57%) patients with valvular involvement at the baseline. The median number of echocardiograms per patient was three (IQR 2), and the median time between the first and last echocardiogram was 57 (IQR 77) months. Comparison of the main valvular findings between first and last echocardiograms is shown in Table 5.

Of note, tricuspid regurgitation was the only echocardiographic finding that evolved in the follow-up (increasing from 40% at initial echo to 62% at last echo; *p* = 0.031). However, severity remained as mild in all of them. Thirty-one (77%) patients with more than one echocardiography performed received anticoagulation after the APS diagnosis was established. The remaining nine (23%) were treated with aspirin.

The evolution of echocardiographic findings did not change regardless of the treatment received. We also found no association between follow-up time, primary or associated APS, and the type and location of thrombosis at the onset of APS with the evolution of valve involvement.

**Figure 1:**
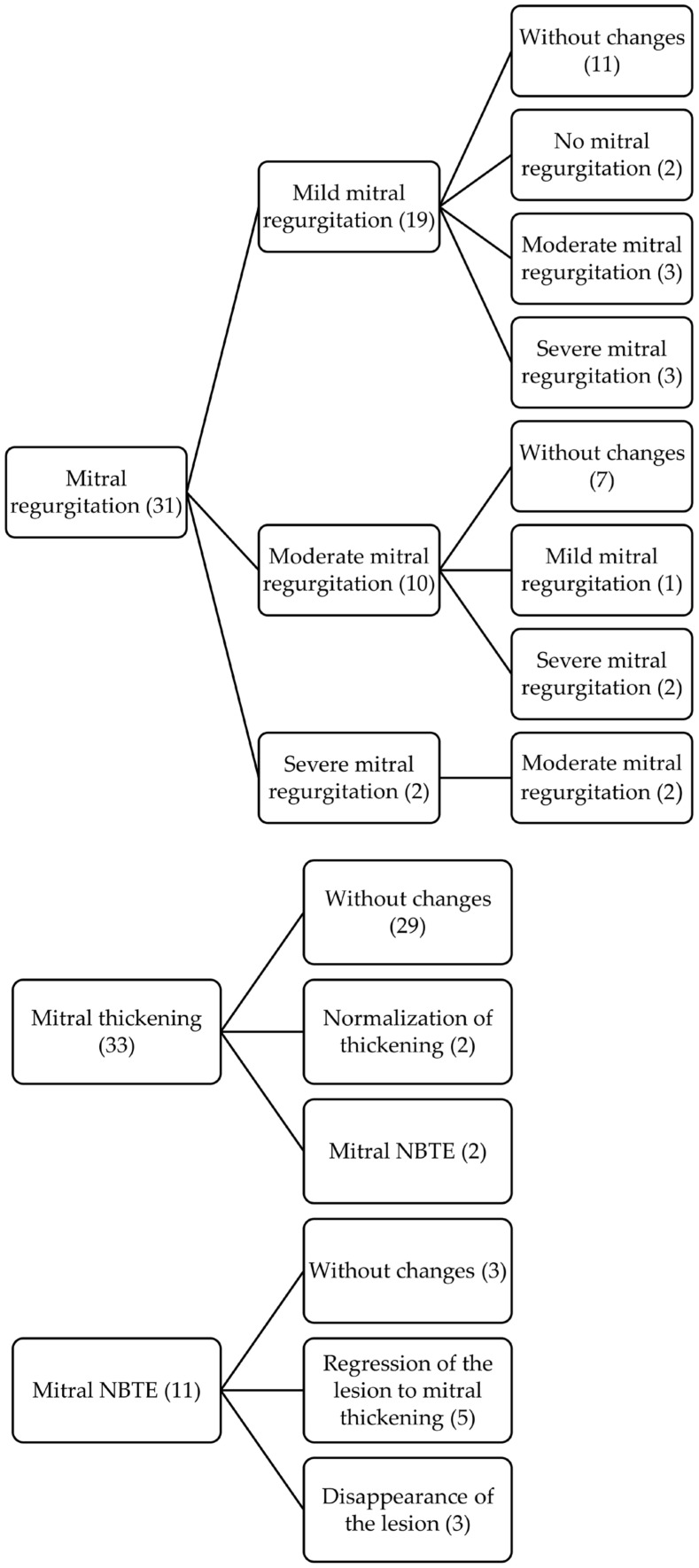
Timeline diagram with evolutive changes of mitral valve involvement.

**Figure 2:**
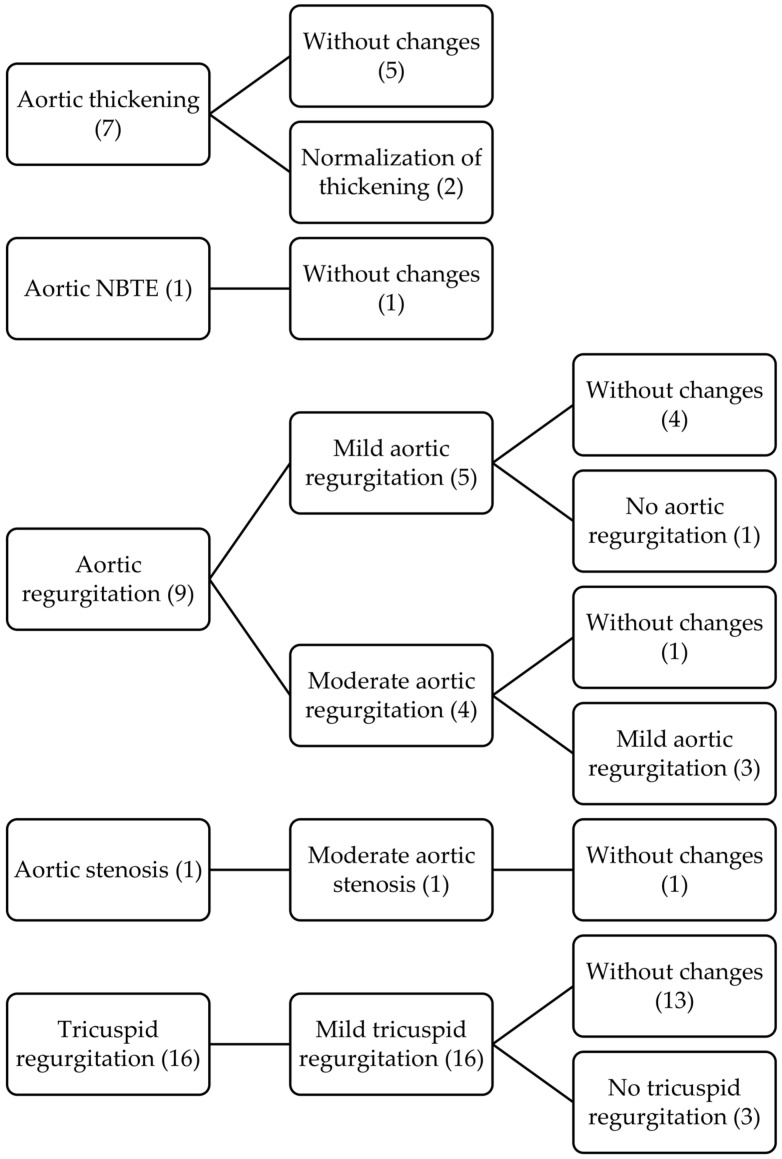
Timeline diagram with evolutive changes of aortic and tricuspid valve involvement.

**Figure 3:**
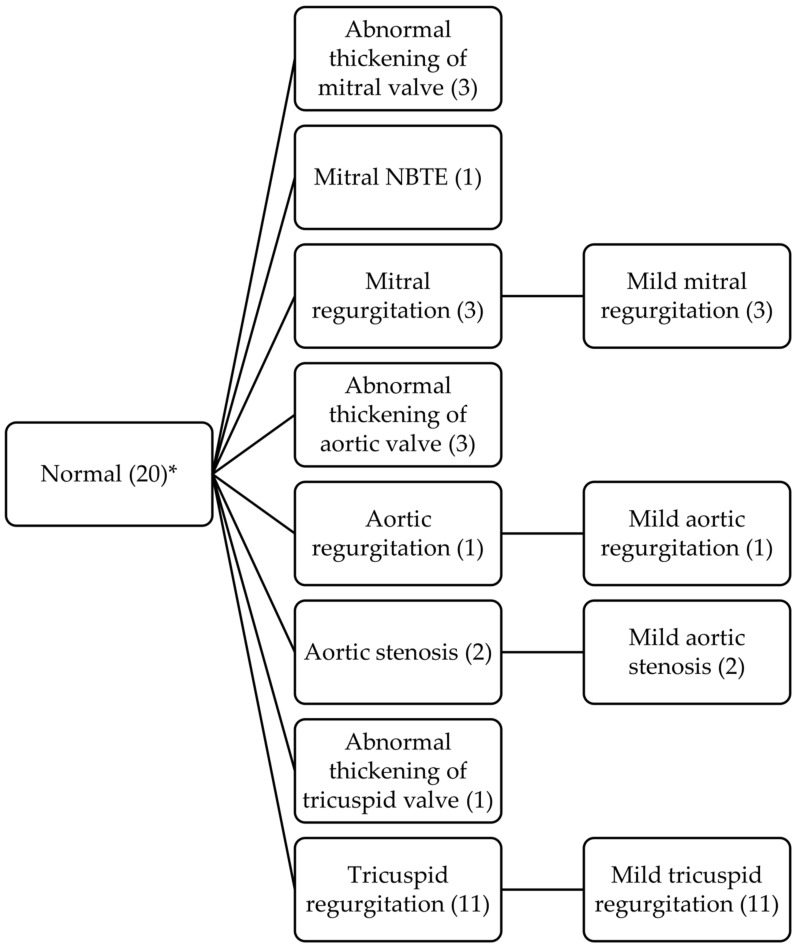
Timeline diagram with evolutive changes of normal valves. * More than one valvular lesion or dysfunction occurred in the same patient. Abbreviations: NBTE: non-bacterial thrombotic endocarditis.

In those patients with more than one echocardiogram, we also describe the evolution of the different morphological and functional lesions, as well as the appearance of new lesions, comparing the findings between the first and last imaging tests performed. The evolutive changes of each lesion are shown in the following timeline diagrams (Figure 1, Figure 2 and Figure 3).

It is noteworthy that of the 11 patients with non-bacterial thrombotic endocarditis on the mitral valve, eight presented regressions of the lesion, disappearing completely in three of them. Of these 11 patients, 10 (91%) received anticoagulation, and only one (9%) was treated with aspirin, who remained with an unchanged thrombotic valve lesion.

## 4. Discussion

In the current APS international consensus of classification criteria, heart valve lesions were included as non-criteria APS manifestations in order to increase their specificity, although their association with aPL is widely recognised [21]. It is important to highlight that valvular involvement has been included in the clinical domain 5 (cardiac valve) in the new proposed ACR/EULAR Classification Criteria for APS with a different weight (two for valve thickening and four for vegetations).

The first cases and series of patients with primary APS and cardiac valve involvement date back to the early years of the 1990s [27,28,29,30,31,32,33], when two-dimensional and Doppler echocardiography studies revealed a 32% to 38% prevalence of valvular defects. Through the use of transthoracic two-dimensional and Doppler echocardiography, several studies [34,35,36,37,38] showed a significantly higher prevalence of valvular defects in SLE patients with aPLs than in those without these antibodies, while another [39] concluded that aCL antibodies either did not play a causative role or were not the only risk factors in the development of cardiac valvular vegetations.

Its therapeutic management is based on the recommendations made by an Expert Group in 2003 [18]. None of the prospective studies conducted has been able to determine clinical manifestations, laboratory features, or different treatments associated with a particular evolution of valvular involvement in APS patients [4,7,13,14,15,16]. More recently, a group of experts has prepared a consensus document with recommendations for the monitoring of these patients [40]. Its high frequency and lack of predictive factors and standardised treatment have led many authors to publish their experience in this pathology, based largely on anticoagulant therapy, immunosuppressive therapy, and valve replacement when operating parameters required it [19,20,41,42,43,44,45,46,47,48,49,50,51,52,53,54,55,56,57].

Valve involvement in APS is characterised by valvular thickening, presence of laminar or warty thrombosis, fibrosis, and low cell infiltration. The isotype IgG of aCL and aβ2GPI and complement have been identified at the subendothelial level, and aCL IgG, IgA, and complement have been objectified at the subendothelial valve [58,59]. Therefore, that suggests a pathogenic hypothesis in which aPL would favour the formation of thrombus in valves by immune complex deposition, promoting valve damage [60].

A recent review on valve disease in autoimmune diseases [61] concluded that the prevalence of valve disease is increased in this group of patients, with thickened and fibrous valves being the most frequent findings, and mitral and aortic regurgitation as the most common sequelae. These three disorders, together with tricuspid regurgitation, are also valvular diseases in our cohort.

Some authors have found a relationship between valve involvement and/or high titres of aCL IgG [7,9,12,15]. Therefore, it has been suggested in a recent meta-analysis [62] that patients with SLE and aCL IgG positive could benefit from a screening echocardiogram. Even the presence of aCL has been associated with an increased risk of prosthetic valve thrombosis [63]. Association between cardiac valve involvement with IgA aPL and the IgG isotype of aβ2GPI against their specific domain 1 (aβ2GPI-D1) [8] or with double positive aCL/LAC patients [10] have been reported. Other studies have tried to demonstrate, with lack of association, the pathogenic role of other antibodies, such as auto-antibodies to Type I and IV collagen that have been described in rheumatic fever and infective endocarditis [64]. We have found a significant association between cardiac valve involvement and LAC, which has been associated with left ventricular diastolic dysfunction in patients with APS [65], while the presence of complement C4 fraction consumption was associated with a decreased risk of valve involvement, opening the door to another possible pathogenic pathway.

The presence of arterial thrombotic events, cardiovascular risk factors, livedo reticularis, migraine, stroke, and epilepsy has been previously described in patients with primary APS with valve involvement [4,7]. Consistent with these findings, we have found a higher frequency of arterial hypertension, a greater number of arterial thrombotic events, stroke, and the presence of livedo reticularis. At the same time, we found fewer venous thrombotic events in patients with cardiac valve involvement. When we have compared patients with moderate-to-severe valvular involvement with those with mild or no involvement, there has remained a higher prevalence of cardiovascular risk factors, as well as hypertension independently, including the presence of arterial thrombosis, stroke, livedo reticularis, and a lower frequency of venous thrombosis, deep venous thrombosis, and pulmonary embolism in those patients with moderate-to-severe valvular involvement.

The higher prevalence of arterial thrombotic events and stroke can be explained in part by the presence of atherosclerosis phenomena [66] attributable both to classic cardiovascular risk factors and the accelerated atherosclerosis itself that occurs in APS [67]. Also, valve involvement would be a risk factor for the development of cerebral embolic events. The presence of livedo reticularis is correlated histologically with endothelial proliferation, not occlusive or inflammatory [68], a phenomenon that has also been associated with early atherosclerosis [69,70]. These findings are included in the concept of APS vasculopathy, defined as proliferation and endothelial dysfunction associated with non-thrombotic manifestations associated with APS. Some authors have postulated the theory that cardiac valve involvement is another form of expression of APS-vasculopathy-affected valvular endothelial cells [4]. This theory would explain the lack of response to anticoagulant and/or anti-inflammatory therapy and its coexistence with arterial thrombotic events and livedo reticularis, two clinical manifestations that in our series also associated with cardiac valve involvement.

In patients with more than one echocardiogram, the appearance or progression of tricuspid regurgitation was the only echocardiographic finding with statistical significance but remained mild in most cases. Previously, none of the different studies [13,14,15,16] could show that the evolution of the echocardiographic findings varied according to the treatment received, and only in a cohort of SLE patients [16] was it found that the presence of APS and disease duration were independent factors for valvular disease progression. We also found no association between the evolution of valve involvement and follow-up time, primary or associated APS, and the type and location of thrombosis at the onset of APS.

The retrospective nature of our series is the main limitation of the study, as well as the inability to obtain all the data for 100% of the patients. In addition, for the classification of the degree of severity of valvular heart disease, a version prior to the 2021 ESC/EACTS Guidelines for the management of valvular heart disease has been used. However, the severity criteria remain the same in both guidelines. Another limitation is to consider valve thickening as cardiac involvement and not as a normal variant, based on the criteria previously defined by our group [7]. In this sense, we wanted to make a comparison between the group with mild valvular involvement and without valvular involvement in relation to the group with moderate and severe valvular involvement, obtaining very similar results. Although we did not find differences between patients with primary APS and those with SLE-associated APS, we did not include SLE patients without associated APS as a control group. This represents another limitation of the study in order to determine the exact role of aPL in heart valve disease. Logically, not all echocardiograms were performed by the same observer, since, in some cases, the initial echocardiogram was performed in another centre, and during 35 years of monitoring, the staff dedicated to performing echocardiography in our centre, of course, has undergone changes. However, the personnel who perform echocardiography are considered experts and are dedicated exclusively to their performance. Another factor to consider is that the review includes patients with primary APS and associated APS, so there may be an overlap of vascular involvement and early atherosclerosis not only for APS but also the associated systemic autoimmune disease, mainly systemic lupus erythematosus. Finally, echocardiographies were performed at the request of the physician in charge of the patient, and they were not done according to a pre-established protocol.

Despite the limitations described above, our results support the hypothesis of the existence of a subgroup of APS patients and cardiac valve involvement with defined clinical characteristics that could be exclusive to patients with moderate-to-severe valvular involvement. Therefore, it could be considered in the future to classify patients with APS into low heart valve risk (without involvement or with mild valve involvement and/or valve thickening) and high valve risk (moderate-to-severe valve involvement and non-bacterial thrombotic endocarditis).

## 5. Conclusions

Valve involvement was very frequent in our cohort of APS patients and was moderate-to-severe in 25% of them. Patients with APS and valvular involvement have their own characteristics with a higher risk of arterial thrombosis and a higher frequency of LAC. In our study, most valve lesions remained stable during follow-up. More prospective studies are needed, but our results support the hypothesis that there may be a subgroup of APS patients with moderate-to-severe valve involvement with its own characteristics that differs from the rest of the patients with APS with mild valve involvement or without valve involvement.

## Figures and Tables

**Table 1:** Demographic characteristics, cardiovascular risk factors and APS related clinical manifestations of patients with APS according to the presence of heart valve involvement detected by echocardiography.

	With Valve Involvement (*n* = 72) N (%)	Without Valve Involvement (*n* = 72) N (%)	*p*
Sex (F/M)	60/12 (83/17)	46/26 (64/36)	0.013
Age at APS diagnosis (years)	41.2 ± 15.1	41 ± 13.9	0.699
Follow-up (months)	115.3 ± 57.6	104.6 ± 61.2	0.213
TTE made at time of APS diagnosis	30 (42)	23 (32)	0.275
Primary APS	48 (67)	57 (80)	0.133
SLE associated APS	22 (30)	14 (19)	0.177
Cardiovascular risk factors ^a^			
Current smoking	21/68 (31)	14/69 (20)	0.112
Ever smoking	16/68 (31)	22/69 (32)	0.260
Arterial hypertension	34 (47)	20 (28)	0.025
Dyslipidemia	14 (19)	17 (24)	0.686
Diabetes mellitus	5 (7)	3 (4)	0.719
Hormonal contraceptives use	7/60 (12)	8/46 (17)	0.196
APS-related clinical manifestations			
Thrombosis	63 (88)	60 (83)	0.638
Arterial	38 (53)	24 (33)	0.028
Venous	23 (32)	36 (50)	0.042
Both	3 (4)	4 (7)	1.000
Peripheral DVT	17 (24)	27 (37)	1.000
Pulmonary embolism	6 (8)	15 (21)	0.057
Ischemic heart disease	4 (6)	3 (4)	1.000
Stroke	27 (38)	15 (21)	0.043
Transient ischemic attack	3 (4)	4 (6)	1.0000
Cerebral silent ischemia	4 (6)	3 (4)	0.439
Epilepsy	6 (8)	3 (4)	0.494
Migraine	10 (14)	8 (11)	0.802
Multiinfarct dementia	3 (4)	2 (3)	1.000
Obstetric morbidity ^b^	37/60 (62)	21/46 (46)	0.681
Livedo reticularis	11 (15)	2 (3)	0.017
Cutaneous ulcers	1 (1)	3 (4)	0.620
Thrombocytopenia ^c^	22 (31)	15 (21)	0.252
Hemolytic anemia	3 (4)	4 (6)	1.000
Renal thrombotic microangiopathy	5 (7)	2 (3)	0.719
Optic neuritis	4 (6)	3 (4)	1.000
Retinal veno-occlusive disease	1 (1)	1 (1)	1.000
Central retinal artery thrombosis	2 (3)	0 (0)	0.497
Central retinal vein thrombosis	1 (4)	1 (1)	1.000
Thrombotic relapse ^d^	22/52 (42)	11/46 (25)	0.858
Hemorrhagic events ^d^	11/52 (21)	9/46 (20)	0.810
Death	9 (12)	1 (1)	0.017

^a^ Including any of those described in the table; ^b^ Including abortions and fetal losses; ^c^ defined as platelet count < 100 × 10^9^/L; ^d^ under anticoagulant treatment. Values of categorical variables are expressed as number and percentage and those for continuous variables are presented as mean ± standard deviation. Abbreviations: APS: antiphospholipid syndrome; DVT: deep venous thrombosis; F: female; M: male; SLE: systemic lupus erythematosus; TTE: transthoracic echocardiography.

**Table 2:** Laboratory features of patients with antiphospholipid syndrome according to the presence of heart valve involvement detected by echocardiography.

	With Valve Involvement (*n* = 72) N (%)	Without Valve Involvement (*n* = 72) N (%)	*p*
LAC	60 (83)	47 (65)	0.021
aCL	56 (78)	56 (78)	1.000
IgG aCL	47 (65)	44/57 (77)	1.000
IgM aCL	26 (36)	21/58 (36)	0.348
aβ2GPI	14/40 (35)	12/43 (28)	0.473
IgG aβ2GPI	10/40 (25)	5/13 (38)	1.000
IgM aβ2GPI	10/40 (25)	8/13 (61)	0.255
aPL profile at APS diagnosis			
Isolated LAC	14 (19)	13 (18)	0.414
Isolated aCL	12 (17)	26 (36)	0.013
Isolated aβ2GPI	1/40 (2)	1/43 (2)	1.000
Double positivity	44 (61)	28 (39)	0.045
Triple positivity	1 (1)	2 (3)	1.000
ANA	43 (60)	58 (81)	0.006
Anti-dsDNA abs	20 (28)	23 (32)	0.716
Anti-Ro abs	5 (7)	8 (11)	0.400
Anti-La abs	3 (4)	3 (4)	1.000
Anti-Sm abs	2 (3)	1 (1)	1.000
Anti-U1-RNP abs	4 (6)	5 (7)	0.745
Low levels of C3	13 (18)	15 (21)	0.834
Low levels of C4	10 (14)	21 (29)	0.042

Including any combination of aPL (LAC plus aCL or aCL plus aβ2GPI or LAC plus aβ2GPI). Abbreviations: aCL: anticardiolipin antibodies; aβ2GPI: anti-β2 glycoprotein I antibodies; ANA: antinuclear antibodies; aPL: antiphospholipid antibodies; APS: antiphospholipid syndrome; LAC: lupus anticoagulant.

**Table 3:** Prevalence of valvular abnormalities on the first transthoracic echocardiography in patients with antiphospholipid syndrome.

Heart Valve Involvement	First Echocardiography (*n* = 72) N (%)
Mitral valve	
Mitral thickening	52 (72)
Mitral regurgitation	49 (68)
Mild	33 (67)
Moderate	10 (21)
Severe	6 (12)
Mitral stenosis	1 (1)
Non-infectious thrombotic mitral endocarditis *	21 (29)
Aortic valve	
Aortic thickening	18 (25)
Aortic regurgitatiom	16 (22)
Mild	9 (56)
Moderate	6 (38)
Severe	1 (6)
Aortic stenosis	2 (3)
Non-infectious thrombotic aortic endocarditis *	5 (7)
Tricuspid valve	
Tricuspid thickening	1 (1)
Tricuspid regurgitation	29 (40)
Mild	29 (100)
Moderate	0
Severe	0

* Also included non-bacterial thrombotic endocarditis that appeared during follow-up.

**Table 4:** Comparison between group with moderate and severe valve involvement versus group without valve involvement and/or with mild involvement or valve thickening without functional impact.

	Significative Valve Involvement N (%) (*n* = 36)	Without or Mild Valve Involvement N (%) (*n* = 108)	*p*
Cardiovascular risk factors			
Arterial hypertension	20 (56)	34 (31)	0.016
APS-related clinical manifestations			
Thrombosis	32 (89)	91 (84)	0.595
Arterial	26 (75)	36 (32)	<0.001
Venous	4 (11)	55 (51)	<0.001
Peripheral DVT	4 (11)	40 (37)	0.003
Pulmonary embolism	1 (3)	20 (18)	0.026
Stroke	21 (61)	21 (18)	<0.001
Livedo reticularis	7 (22)	6 (5)	0.019
Laboratory features			
LAC	33 (92)	74 (68)	0.007
aPL profile at APS diagnosis			
Isolated LAC	5 (11)	22 (20)	0.137
Isolated aCL	4 (11)	34 (31)	0.346
Isolated aβ2GPI	0 (0)	2 (2)	1.000
Double positivity	27 (75)	49 (44)	0.032
Triple positivity	1 (3)	2 (2)	0.486

Includes any combination of aPL (LAC plus aCL or aCL plus aβ2GPI or LAC plus aβ2GPI). Abbreviations: aβ2GPI: anti-β2 glycoprotein I antibodies; aCL: anticardiolipin antibodies; aPL: antiphospholipid antibodies; APS: antiphospholipid syndrome; LAC: lupus anticoagulant.

**Table 5:** Comparison of heart valve involvement between first and last echocardiograms.

Initial Echocardiogram	Patients (*n* = 40)	Follow-Up Echocardiogram	Patients (*n* = 40)	*p*
**Mitral valve**		**Mitral valve**		
Thickening	33 (82)	Thickening	32 (80)	0.711
Regurgitation	31 (77)	Regurgitation	32 (80)	0.628
Mild	19/31 (61)	Mild	15/32 (47)	
Moderate	10/31 (32)	Moderate	12/32 (37)	
Severe	2/31 (6)	Severe	5/32 (16)	
**Aortic valve**		**Aortic valve**		
Thickening	7 (17)	Thickening	8 (20)	0.660
Regurgitation	9 (22)	Regurgitation	10 (25)	0.660
Mild	5/9 (56)	Mild	9/10 (90)	
Moderate	4/9 (44)	Moderate	1/10 (10)	
Severe	0/9 (0)	Severe	0/10 (0)	
Stenosis	1 (2)	Stenosis	3 (7)	0.323
**Tricuspid valve**		**Tricuspid valve**		
Thickening	0 (0)	Thickening	1 (2)	0.323
Regurgitation	16 (40)	Regurgitation	24 (62)	0.031
Mild	16/16 (100)	Mild	24/24 (100)	
Moderate	0/16 (0)	Moderate	0/24 (0)	
Severe	0/16 (0)	Severe	0/24 (0)	

## Data Availability

The datasets generated and/or analyzed during the current study are available from the corresponding author on reasonable request.

## Supplementary Materials

The following supporting information can be downloaded at: https://www.mdpi.com/article/10.3390/jcm12082996/s1, Table S1. Type of valve involvement between patients with primary APS and patients with SLE-associated APS.
