# Supplementary material for: Heart Valve Involvement in Patients with Antiphospholipid Syndrome: A Long-Term Follow-Up Study of a Single Centre
Source: J Clin Med. 2023 Apr 20;12(8):2996. doi: 10.3390/jcm12082996 (PMC10145646; doi:10.3390/jcm12082996)
Supplement: Supplementary file 1 [file jcm-12-02996-s001.zip › jcm-2341270-supplementary.pdf]

Supplementary Table S1. Type of valve involvement between patients with primary APS and patients with SLE-associated APS.

Valvular involvement	Primary APS (n=48) N (%)	SLE-associated APS (n=22) N (%)	p
**Mitral valve**			
Thickening	37 (77)	15 (68)	0.557
Regurgitation	35 (73)	12 (54)	0.172
Stenosis	1 (2)	0 (0)	1.000
NITE	16 (33)	5 (23)	0.415
**Aortic valve**			
Thickening	11 (23)	6 (27)	0.762
Regurgitation	9 (19)	7 (32)	0.222
Stenosis	1 (2)	1 (4)	0.519
NITE	2 (4)	2 (9)	0.585
**Tricuspid valve**			
Thickening	0 (0)	1 (4)	0.304
Regurgitation	23 (48)	5 (23)	0.069

Abbreviations: NITE: non-infectious thrombotic endocarditis.
